# “Vegan Teachers Make Students Feel Really Bad”: Is Teaching Sustainable Nutrition Indoctrinating?

**DOI:** 10.3390/foods11060887

**Published:** 2022-03-21

**Authors:** Alina Weber, Laura Linkemeyer, Lena Szczepanski, Florian Fiebelkorn

**Affiliations:** Didactics of Biology, Department of Biology and Chemistry, Osnabrück University, Barbarastrasse 11, 49076 Osnabrück, Germany; llinkemeyer@uni-osnabrueck.de (L.L.); lena.szczepanski@uni-osnabrueck.de (L.S.); florian.fiebelkorn@uni-osnabrueck.de (F.F.)

**Keywords:** education for sustainable development (ESD), sustainable nutrition, biology teachers, teacher training, indoctrination, dietary choices

## Abstract

The transformation toward more sustainable food choices may be supported by an education for sustainable nutrition. In schools, biology teachers play a key role in educating students as sustainability change makers, as biology lessons provide various opportunities to deal with ESD-topics such as sustainable nutrition. Teachers’ classroom practices may be influenced by their personal choices regarding sustainable nutrition. Additionally, students may see their teachers as role models for sustainable behavior. This presents the risk of students adopting teachers’ beliefs and behaviors without reflection. Teaching sustainable nutrition is therefore in potential conflict with indoctrinating young people toward sustainable diets. To date, no studies have addressed the perceived risk of indoctrination in the context of teaching sustainable nutrition in relation to teachers’ personal beliefs, behaviors, and teaching practices. Therefore, this study explored whether biology teachers themselves perceive a risk of indoctrination when teaching sustainable nutrition, and what methods they use for teaching it in a non-indoctrinating way. For this purpose, we conducted semi-structured interviews with seven in-service biology teachers from high schools in Germany. Data for this explorative qualitative study were collected from July to October 2021 using convenience sampling. These were analyzed by qualitative coding and content analysis. The preliminary results of this study show that participants recognize a high risk of indoctrination when teaching sustainable nutrition, primarily due to their own teaching actions, such as deciding whether to reveal their own dietary choices to students. While some participants believed teachers must be restrained, others thought that open communication about personal choices could benefit student decision-making skills. In terms of avoiding indoctrination when teaching sustainable nutrition, participants advocated for student-centered and multi-perspective teaching approaches. Based on the findings, initial implications for further research and teacher training are discussed.

## 1. Introduction

The current food system and people’s individual eating habits are receiving growing attention in the realm of sustainable development, as they have a significant impact on the health of people and the planet [1,2,3]. Unsustainable eating habits are partly responsible for global challenges such as biodiversity loss, climate change, social and financial injustice, as well as food-related health risks and non-communicable diseases such as overweight and obesity [4,5]. Therefore, the concept of a “planetary health diet” [3] connects the perspectives of health and sustainability. It addresses how nutrition choices can transform the global food system while simultaneously reducing non-communicable diseases and protecting the planet [6,7]. The transformation of the current food system and personal dietary styles is also crucial for achieving the United Nations’ 17 Sustainable Development Goals (SDGs), such as SDG 2, “Zero Hunger,” or SDG 12, “Responsible Consumption and Production” [8]. For achieving the SDGs, and in particular target 4.7, “Quality Education,” education for sustainable development (ESD) is necessary [8]. One of the school subjects in which ESD can be meaningfully embedded—and used to help students develop evaluation competencies and critical thinking skills—is biology. Topics of sustainable development are particularly suitable for implementing ESD and promoting evaluation competencies in biology lessons at all grade levels [9]. More specifically, for implementing ESD in biology lessons, the topic of sustainable nutrition offers great potential, as nutrition is a daily behavior that is linked to decision-making behaviors [10]. Within the scope of an education for sustainable nutrition, ecological, economic, social, health, and cultural concerns should be considered [11]. Thus, the topic provides multidimensionality and relevance to everyday life [12]. However, sustainable nutrition is not mentioned as an example topic in German school curricula due to schools’ competency-based approach. Therefore, the onus is on biology teachers for implementing sustainable nutrition into school biology lessons and preparing suitable learning situations [9,13,14]. Their ways of thinking and understanding can affect teaching activities and teaching motivations [15,16], inevitably altering students’ behavior and attitudes toward ESD [14]. Thus, biology teachers are responsible for evaluating different sources of teaching materials and acting responsibly when educating their students on sustainable nutrition, as they are at risk of indoctrinating their students [17]. Especially when dealing with ESD topics such as sustainable nutrition, biology teachers should enable students to make their own informed decisions [18,19]. This is one central aim of evaluation competencies that must be fostered in biology lessons [19,20]. Enabling students to develop evaluation competencies is important in areas of sustainable nutrition and especially regarding choices of meat consumption, meat alternatives, vegetarianism, and veganism [21]. Studies show that even experienced teachers have difficulties maintaining their professional neutrality in terms of separating their personal opinions and preferences from teaching [22].

Student biology teachers have been shown to strongly favor eating sustainably in their daily lives [23,24], and their personal intentions to eat sustainably can lead to an intention to teach this topic in school [25]. Due to their potential role model status, regarding sustainable behavior, this relationship between private choices and teaching behavior should be considered when biology teachers address sustainable nutrition in the classroom. Thus, biology teachers may unwittingly provide their students direction aligned with their own attitudes and actions [26]. According to Håkansson [27], teachers with especially strong opinions about controversial, value-laden educational topics such as sustainable nutrition should remain almost neutral to educate their students rather than to indoctrinate them.

### 1.1. Teaching Sustainable Nutrition versus Risk of Indoctrinating

In general, “indoctrination” is defined as manipulating individuals or entire groups of society by psychological means in order to form a particular opinion or attitude [28]. Regarding teaching contexts, indoctrination means to teach content manipulatively and regardless of evidence to the contrary; thus, it is one-sided, with the intention of changing students’ attitudes and behaviors [29,30].

In terms of existing educational standards and curricula, ESD aims to engage critically with moral values and norms in the classroom. However, these moral values and norms should not be indoctrinated [19,20]. For this purpose, biology lessons should promote communication and evaluation competencies [20]. In the context of evaluation competencies, students should learn to adopt different perspectives and develop multi-perspective thinking in order to participate in social discourse, which may sometimes be controversial [19,20]. These specifications imply that teachers should not push their students in a direction of particular thoughts or values.

Along these lines, in 1976, the Beutelsbach Consensus, which stems from political education, adopted the prohibition of indoctrination in education [31,32]. The consensus includes firm didactic guidelines for teachers: (1) It prohibits overwhelming a student with an opinion (“overwhelming prohibition”), (2) demands multi-perspective approaches and presentations (“controversy requirement”), and (3) includes enabling students to analyze existing situations (e.g., in this context, consequences of non-sustainable food productions and dietary styles) and seek ways to influence them in terms of their own interest (“orientation of interest”) [31,32].

In the context of this study and according to the “overwhelming prohibition” guideline, indoctrination is defined as an action in which teachers illicitly overwhelm their students with the teachers’ own attitudes and opinions. Due to the “controversy requirement,” teaching sustainable nutrition demands multi-perspective approaches [19,20]. With regard to the required “orientation of interest,” biology lessons on sustainable nutrition should offer students opportunities to assess the sustainability of their own dietary styles in the transition toward a more sustainable society.

Indoctrination when teaching sustainable nutrition involves one-sided teaching approaches or materials that support the teachers’ beliefs. As an example, a vegan biology teacher may choose teaching materials that focus on the benefits of a vegan diet, without considering counterarguments. These materials would solely give information on the drawbacks of meat consumption and the benefits of vegan diets, but hide information on the benefits of meat consumption and limitations of veganism. Consequently, biology teachers who indoctrinate their students about sustainable nutrition during biology lessons prevent them from forming self-determined opinions about their diet [31]. Accordingly, teachers need to pay attention to controversial positions in the classroom and empower students to make their own decisions with the help of lessons on sustainable nutrition [31,32]. This can be done, for example, by using the method of data-based decision making, with which the students will evaluate the sustainability of vegan diets compared to an omnivorous diet [33].

Thus, indoctrination is incompatible with the role of the teacher in a democratic society and the aims of ESD and evaluation competencies [31,32]. Both ESD and education for sustainable nutrition seek to avoid indoctrinating students, but aim to educate students to be able to think and act responsibly in order to shape the future sustainably [18,34].

In principle, sustainable nutrition is a suitable topic to meet the principles of the Beutelsbach Consensus and to ensure that students form their own decisions about sustainable nutrition. It is a socially controversial topic, as many people discuss the merits of a vegan diet for children and adolescents, as well as the healthiness of controversial foods such as red meat or dairy products. Related issues also make this topic controversial, such as the dilemma between industrial animal husbandry, consumer requirements, and economic consequences for farmers.

### 1.2. Aims of the Present Study

As presented above, teaching sustainable nutrition offers some risk of indoctrination if not handled carefully [27]. There is a need to examine whether biology teachers perceive a risk of indoctrination when addressing sustainable nutrition in biology lessons. So far, the issue of a potential risk of indoctrination has solely been explored from a student perspective, in relation to biology teachers’ authenticity [35,36]. However, to our knowledge, no study has yet examined the perceptions of in-service biology teachers related to the potential risks of indoctrination when teaching sustainable nutrition, nor has any study shed light on teaching approaches to avoid indoctrination in biology lessons.

The present study provides a glimpse of the thinking and argumentation structures of biology teachers in Germany toward teaching sustainable nutrition and the potential risk of indoctrination. This research is relevant, as biology teachers are in a key position to decide how to teach sustainable nutrition. Due to the multiplier effect, this decision can in turn affect students’ thinking. The findings can then also be transferred to other contexts, samples, and countries. Based on the findings of this study, approaches to biology teacher training can be enhanced. Thus, the following research questions were formulated:

- Research Question 1: Do in-service biology teachers perceive a risk of indoctrination when teaching sustainable nutrition? If so, in what ways?
- Research Question 2: To what extent would in-service biology teachers share their own dietary style with their students, and do they see a risk of indoctrination in sharing this personal information?
- Research Question 3: What approaches would in-service biology teachers use to teach sustainable nutrition and avoid indoctrination in biology lessons?

## 2. Materials and Methods

### 2.1. Study Design and Sample

To recruit the participants, an email was initially sent to the respective school principal. This email contained a formal letter requesting permission to collect data with biology teachers at the respective high school. The letter outlined the substantive interest of the study and the plans for data collection. The school principals then asked for volunteer participants among their biology teachers. The interested teachers contacted the first or third author by email. Thus, participants were recruited using convenience sampling. They were included in the study based on the two criteria that they teach at high schools in Lower-Saxony or North Rhine-Westphalia and have biology as one of their teaching subjects. Teachers of other school types or without having biology as subject were excluded from this study. In addition, we aimed to acquire both female and male teachers with different years of teaching experience. In total, seven in-service biology teachers from Osnabrück and the Münsterland region were interviewed (*N* = 7; Teaching experience: *M* = 9.4 years, *SD* = 8.81; 57% female). Thus, participants came from the federal states of North Rhine-Westphalia and Lower Saxony. These federal states use different curricula for teaching biology. Information on the participants is presented in Table 1. All participant names have been replaced by pseudonyms to maintain anonymity. Two participants indicated that they followed an omnivorous diet, two were vegetarian, and there was one pescatarian, one flexitarian, and one vegan person (Table 1). According to the relatively small sample size of seven biology teachers, the present study must be characterized as exploratory.

Data collection took place from July to October 2021. All interviews were conducted by the third author. Due to contact restrictions imposed by the COVID-19 pandemic, the interviews were conducted using BigBlueButton videoconferencing software from Osnabrück University [37]. The study was carried out in accordance with the national and institutional guidelines, the Declaration of Helsinki, the German Research Foundation, and the American Psychological Association. Anonymity of the participants was guaranteed, and the participation was voluntary. All participants had the chance to decline participating in the study at any time and without any consequences. The participants previously signed a declaration of consent that contained information on the main topic of the interview, the study procedure, as well as the recording and further processing of the data. As the research had no medical background, it involved no risks to our participants. Moreover, the research assessed no sensitive personal data. In consequence, an ethics approval was not required.

For optimization of interview questions and the interview procedure, a pilot interview was held in advance with another participant. The pilot interview was not included in the final sample. The audio tracks were recorded using a digital audio recorder (Olympus WS-852). The duration of the interviews ranged from 15 to 25 min (*M* = 18.40 min; *SD* = 3.10). This time specification includes only the main phases of the interviews, not the phases before the start and after the end (e.g., welcoming, small talk, information on the interview procedure, and filling out a short questionnaire). The interviews were conducted in German and the statements of the participants were translated into English for the purpose of this paper.

### 2.2. Interview Procedure and Study Design

The qualitative research design is based on using a semi-structured interview guide developed by Linkemeyer [38] to address the research questions. The guide served as an orientation for the interviewer and was used to ensure comparability of the data. Because the guide was semi-structured, it allowed the interviewer and participants to develop discussions, while enabling participants to express their thoughts in a flexible way [39].

**Figure 1:**
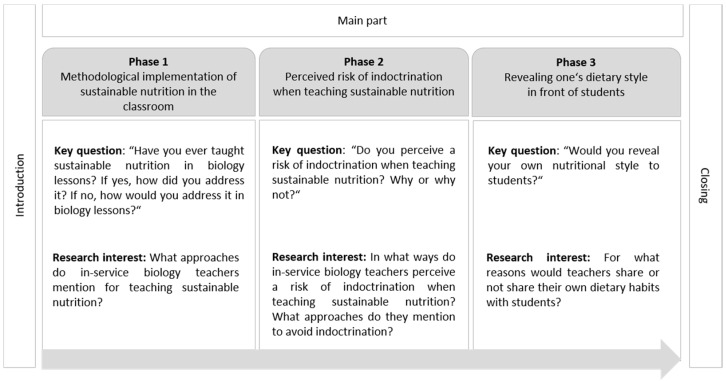
Key Questions and Research Interests of the Three Main Phases of the Interviews.

In general, the interview was divided into three parts: (1) an introduction to the study, (2) the main part, and (3) a closing, which included a short questionnaire. The main part of the interviews consisted of three phases relevant for answering the three research questions (Figure 1). The complete interview guide can be obtained from Supplementary Material File S1. In Phase 1, previous teaching experiences with education for sustainable nutrition in biology lessons, as well as approaches to address this topic in the classroom, were assessed. In Phase 2, participants were asked if—and in what ways—they perceive a risk of indoctrination when teaching sustainable nutrition. Following this, possible teaching methods to avoid indoctrination were discussed. In Phase 3, participants were asked whether they would reveal their own dietary style to their students. At the end of the interview, a short questionnaire was filled out with the respective participant. The questionnaire was used to collect the dietary style, gender, teaching experiences, and opinions toward the importance of dealing with ESD and sustainable nutrition in schools (Table 1).

### 2.3. Data Analysis

**Figure 2:**
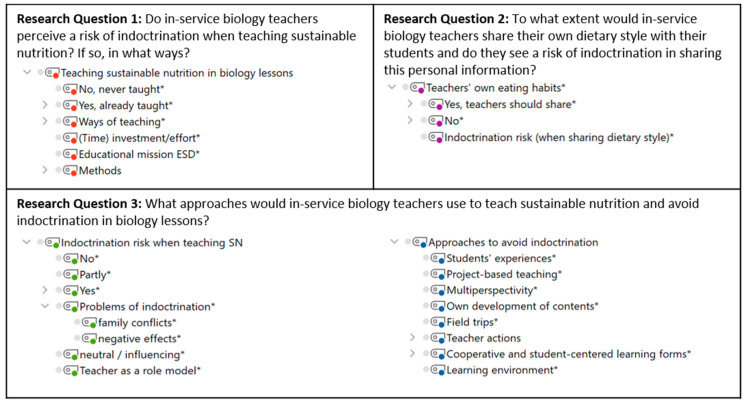
Category Systems for Answering the Research Questions. * Inductively coded categories. ESD = education for sustainable development; SN = sustainable nutrition.

Audio recordings were transcribed according to the guidelines of Dresing and Pehl [40]. The transcripts were analyzed in MAXQDA [41] using qualitative content analysis following methods described by Mayring [42]. Following the research questions and three phases of the interview, a deductive category system was created (Figure 2) using superordinate categories: (1) “teaching sustainable nutrition in biology lessons”, (2) “indoctrination risk when teaching sustainable nutrition”, (3) “approaches to avoid indoctrination”, and (4) “teachers’ own eating habits”. Under the heading of each of these superordinate categories were more specific inductive codes, which were derived from the participants’ statements (Figure 2).

To improve coding quality, a second person coded all statements relevant for answering the research questions. To assess the level of agreement between both coders, the intercoder agreement was calculated in MAXQDA according to methods described by Brennan and Prediger [43]. A Kappa value of 0.78 revealed a substantial intercoder agreement [43].

## 3. Results

Even though participants noted that sustainable nutrition is not explicitly mentioned in subject-specific or school curricula, four of the seven teachers said they have previously taught sustainable nutrition or some aspects of it, such as animal husbandry, that could be considered part of the overarching topic. Two of the teachers said they taught sustainable nutrition because of their personal interest in the topic, and two other participants taught it in order to confront students with their own dietary styles. In addition, three of them indicated that they have engaged with students on sustainable nutrition outside of regular biology lessons, such as in special subjects or project work. One teacher, Mrs. Williams, described her belief in the necessity of teaching sustainable nutrition as part of biology teachers’ educational mission on ESD:

“[It is our mission as teachers] to educate with ESD in mind—[…] how we can deal with our future […] in order to shape it sustainably. And, in the end, an essential part is sustainable nutrition. […] Nutrition plays a major role in ESD, because it is something we can always do on our own as individuals.”

(Mrs. Williams, 9)

A summary of the main findings for each of the seven participants in regard to the three research questions can be obtained from Table 2.

### 3.1. Risk of Indoctrination When Teaching Sustainable Nutrition

Six of the seven participants perceived a clear risk of indoctrination when teaching sustainable nutrition. However, the participants assessed this risk differently, either in relation to themselves as teachers, to teachers in general, or to learning materials.

Most participants maintained that teachers are responsible for avoiding indoctrination, and they saw the risk of indoctrination as directly related to the teacher. They expressed that the teachers themselves are primarily responsible for indoctrinating their students (or not), and consequently, they themselves can also contribute to avoiding it. As Mr. Wood explained:

“It depends a lot on how you deal with [sustainable nutrition] as a teacher. I think that indoctrination can be prevented by […] promoting the students’ ability to evaluate [certain contexts and situations]. […] That means that indoctrination can indeed take place through teachers, but I think that they are often aware of that risk and should pay attention to their pedagogical principles.”

(Mr. Wood, 37)

Three participants related a higher indoctrination risk to teachers who themselves behave in environmentally friendly ways or explicitly eat sustainably, and subsequently teach sustainable nutrition in the classroom: “Vegan teachers […] make students feel really bad” (Mr. Evans, 61). They indicated their belief that vegan teachers implicitly or explicitly create a feeling of guilt in students about not behaving or eating sustainably, consequently having a negative impact on their health and the environment. The idea of evoking a sense of guilt in students has its own problems, such as the fact that most students are not in control of their food choices:

“[Many] students do not have the ability to go shopping for themselves. […] There is, of course, a great risk that you could make the students look very bad through […] your teaching and your statements, and you could also give them a very bad conscience.”

(Mr. Evans, 61)

Mrs. Williams stated that teachers who are especially environmentally conscious are personally responsible for not indoctrinating their students. At the same time, she mentioned possibilities for avoiding the potential risk of indoctrination:

“I think it is difficult when […] a teacher tries to live environmentally consciously and to eat as regionally and seasonally as possible or without meat. […] [As a vegan or vegetarian teacher] I think it is sometimes difficult […] to hold back [in the classroom] when it comes to animal husbandry or something like that. But I think it’s ok if you consciously decide to shed light on different perspectives and always integrate this [into your teaching].”

(Mrs. Williams, 35)

Mr. Smith was the only teacher who clearly stated that teaching materials themselves might impose a risk of indoctrination. However, at the same time, he held teachers accountable for not indoctrinating and advocated that they should be responsible for selecting, using, and adapting the teaching materials.

“Over the years, […] [biology teachers] have been supplied with materials […] free of charge. For example, from the dairy industry or from agricultural interest groups. […] There is already filtered information. [The materials] do not show the whole range of a sustainable food economy and there are opportunities for indoctrination. […] But you don’t have to be subject to that. […] It is a question of the individual teacher to deal with it […] consciously and to say, ‘No I do not use the materials that way, I prepare them differently or I use my own materials.’”

(Mr. Smith, 37)

Mrs. Robinson stated that she thinks there is risk of indoctrination in connection to teachers functioning as role models, especially for young students:

“I think [whether you indoctrinate or not] always depends on the teacher, because you […] [automatically] serve as a role model for the younger students, especially in the fifth grade.”

(Mrs. Robinson, 37)

Mrs. Williams and Mr. Evans both said they perceived the risk of indoctrination as something that is common in teaching contexts, especially when teaching younger students. As Mrs. Williams explained,

“I think that somehow a teacher probably always influences students. […] Of course, it may be that I influence a student with my opinion, but that is not intentional […] and as long as you hold back, I think it is okay.”

(Mrs. Williams, 39)

Mrs. Robinson saw a problem with teaching sustainable nutrition because it could lead to conflicts with parents, since it addresses aspects of the students’ and their parents’ private-sphere behaviors in terms of food selection and nutrition. She considered teaching sustainable nutrition as something similar to teaching sensitive topics, such as sex education:

“As a teacher, I would be careful to present [sustainable nutrition] as objectively and neutrally as possible, […] just as in sex education […] or contraceptives, which is also similar, as this really affects the private sphere [of the students]. [Teachers] should […] respect the natural right of education of the parents and [be careful] that the parents do not feel attacked somehow.”

(Mrs. Robinson, 37)

### 3.2. Revealing Teachers’ Own Dietary Style to Students

In Phase 3 of the interviews, participants were asked whether teachers should reveal their own dietary style to their students. In general, most of them were in favor of announcing their own dietary style in the classroom when teaching sustainable nutrition. Three of the teachers said they would only reveal this information if students asked. As Mr. Evans explained:

“If [the students] ask me, sure. If they don’t ask me, I wouldn’t say anything [about my own dietary style]. I would also explain why [I am vegetarian]; you are also a [role model for the students]. […] [As a teacher you] should build up a personal relationship [with your students]. Accordingly, [revealing the own dietary style to students] […] is completely legitimate in my eyes. It’s just like when you ask about political positions, you can also disclose them and explain them, and the student can still have a different opinion.”

(Mr. Evans, 67)

Mr. Evans also stated that revealing one’s dietary style is part of an open and personal relationship with the students. Mr. Wood went one step further and declared that revealing one’s own dietary style is part of teachers’ authenticity, which fosters the students’ own decision-making competence:

“I personally would have no problem in sharing [my own dietary style to students], […] because for me it is also part of [teachers’] authenticity. But I can understand every teacher who doesn’t [reveal the own dietary style to students]. But then [the teachers] do not have to complain if it’s harder for the students to go their own way [in terms of a self-determined diet].”

(Mr. Evans, 67)

In contrast, Mrs. Taylor, the only vegan teacher in this sample, perceived a high risk of indoctrination due to her own dietary style:

“I am vegan and very convinced of my way of life. I really have to make sure that I remain objective and don’t dictate my opinion to the students.”

(Mrs. Taylor, 53)

Mrs. Wilson shared an ambivalent attitude about disclosing her own dietary style to her students. She perceived a risk of indoctrination specifically due to teachers’ function as role models: students might adopt the dietary style of their role model teachers without reflection, which Mrs. Wilson considered as indoctrination. Furthermore, she perceived that the dietary style of teachers can lead to social pressure within the learning group, if the teachers’ dietary style is perceived as the correct and desirable behavior by the students. As she explained:

“I think [revealing my own dietary style to students] is a double-edged sword. Of course, as a teacher you are a role model in a certain way and if I […] tell the students that I consciously eat less meat for [different] reasons, and the other students all have their sausage as their lunch and then they say at the end ‘Oh, but Mrs. [Wilson] said that you are not really allowed to do that,’ then we are already back in the area of indoctrination, where I, of course, have a little more power of speech […] than the individual student. […] That would probably depend on the class in which you [reveal your own dietary style]. […] For example, if you stimulate a discussion about how […] [to] implement sustainable nutrition in our everyday lives, and […] the teacher is just one of many. But I would not stand there as […] a single example that has a normative character.”

(Mrs. Wilson, 45)

### 3.3. Methodological Implementation of Sustainable Nutrition and Avoiding Indoctrination

Primarily, the teachers said they would teach sustainable nutrition as part of teaching nutritional physiology at lower grades of high school. None of the participants said they embedded sustainable nutrition in teaching units about sustainability. Moreover, four participants referred to teaching sustainable nutrition in an interdisciplinary context, such as in connection to geography lessons. In addition, Mrs. Wilson, Mrs. Robinson, and Mr. Smith all said they aim to provide students with practical skills, in terms of preparing their own sustainable meals in special subjects or project work.

Some of the participants mentioned specific teaching topics of sustainable nutrition, such as agriculture, meat alternatives, or health and environmental consequences of food consumption. Most of them suggest that teachers focus on basic terms and concepts of sustainable nutrition, such as the concept and definition of sustainability, the dimensions of sustainable nutrition, the multidimensional consequences of peoples’ dietary styles, as well as the comparison of healthy and sustainable diets. Two teachers explicitly mentioned fostering evaluation competencies as an explicit learning goal when addressing sustainable nutrition in the classroom.

When talking about methods for teaching sustainable nutrition and simultaneously avoiding indoctrination, the participants mostly mentioned “methods with high student involvement” (Mr. Wood, 43). Generally, participants cited a great variety of methods, but primarily focused on cooperative and student-centered activities, such as group work and various types of discussions. As Mrs. Wilson explained,

“[It is important] that one chooses rather cooperative forms of teaching [sustainable nutrition]. Then, of course, one will again escape one’s own indoctrination tendency a little bit.”

(Mrs. Wilson, 39)

Five of the seven participants talked about implementing multi-perspectivity in the classroom when teaching sustainable nutrition. Mrs. Williams explained that these perspectives are important so that students can make their own informed decisions:

“It must […] become clear to the students that you can’t just look at [sustainable nutrition] one-dimensionally [in the sense that we all have to eat sustainably]. Thus, looking at [sustainable nutrition] from different perspectives is important, because it is not always black and white. And we [as teachers] are required to illuminate different perspectives [for our students]. […] In the sense of good teaching, […] you [should] create diversity and then students should form their own opinion based on what we have worked out.”

(Mrs. Williams, 27)

With regard to multi-perspectivity, the same teacher talked about the necessity of interaction and making different information sources available to students:

“I think it is very important that you […] [teach sustainable nutrition] with a large variety of methods, so that you don’t just give texts, but also look at videos and so on. So that you also see that it is all very diverse, and so that you look at the different perspectives. I would avoid doing exclusively frontal teaching, individual work or partner work. I think it is very important to […] interact with each other.”

(Mrs. Williams, 27)

To avoid indoctrination through the usage of selected methods, Mr. Evans advised that students could present their informed ideas through a debate-style learning exercise:

“One could […] have a debate at the end of a lesson [on sustainable nutrition]. [One of the following topics could be the basis for such a debate:] Should a veggie day be introduced in Germany? […] If you have such a debate, pro and contra arguments are elaborated and […] if the teacher chooses balanced teaching material, [the pro and contra arguments] […] have to be [roughly equally distributed]. […] [When pro and contra arguments are roughly equally distributed], […] it is given that there is no indoctrination. Because […] [the students and the teacher] capture [the topic of the debate] completely.”

(Mrs. Williams, 27)

When participants were asked about lesson designs for avoiding indoctrination, they focused on fostering students’ decision-making competencies, which would enable supportive learning environments and teacher actions, such as holding back their own opinions and being able to respect other opinions. According to participants, students should develop the content on their own, and the teacher should perform more as a moderator, restraining their personal beliefs. For example, Mr. Evans described that moderating the role of teachers might avoid indoctrination:

“I would first take a back seat as a teacher, so that the students can work it out for themselves with materials that are balanced and not one-sided. […] In the end, everyone is free to eat what he or she would like to eat. [The teacher should explicitly] point this out. There may be students who are already vegetarian. Teachers should also make sure that it is a well-mannered learning environment.”

(Mrs. Williams, 27)

Additionally, the participants indicated that there are several factors that influence lesson planning for teaching sustainable nutrition. Primarily, they considered the students themselves as one important factor for lesson design. One participant noted that the social and financial backgrounds of students are relevant factors that should be considered when teaching sustainable nutrition. Students’ backgrounds might make it difficult or impossible for them to buy regional and organic products, due to financial constraints. This could especially lead to conflicts if teachers, as role models, advocate eating sustainably; when students carry this message home, parents must justify their reasons for this ideal diet simply not being possible. Moreover, as the above participants expressed several times, lesson design depends on the students’ age and ability to decide on their own meals.

Going beyond student-related and socio-financial factors, participants associated teaching sustainable nutrition with time and organizational hurdles, as the topic of sustainable nutrition is not explicitly addressed in the school curricula. Thus, they perceive it as extra work and effort. Mr. Smith discussed this as a barrier to teaching this topic:

“[My] experience is that [teaching sustainable nutrition] is very time-consuming. […] There are a lot of organizational reasons that counteract […] [teaching sustainable nutrition]. But it is of course the case that you can also give students a lot to take with them on their way [to a more sustainable way of living].”

(Mrs. Williams, 27)

## 4. Discussion

In line with the officially declared suitability of sustainable nutrition as a learning field for ESD [10], teaching this topic was perceived by the biology teachers who participated in this study as an important and relevant topic on the way toward a sustainable transformation of our society. Moreover, the participants perceived sustainable nutrition as a topic that every student can actively be involved in and contribute to. The statement that every individual can make a positive contribution to sustainable nutrition can be supported by prior findings showing that young people perceived a relatively high consumer effectiveness related to sustainable nutrition [44,45,46].

In the following section, the research questions are addressed in the context of participants’ overall responses and discussions. Section 4.1 presents a discussion of the first research question, whether biology teachers perceive a risk of indoctrination when teaching sustainable nutrition. Section 4.2 presents the second research question, whether and how biology teachers share their personal dietary style with their students, and if they perceive a risk of indoctrination in this practice. Finally, Section 4.3 addresses the third research question, concerning what approaches biology teachers would prefer to take to teach sustainable nutrition and avoid indoctrination.

### 4.1. Research Question 1: Perceived Risk of Indoctrination When Teaching Sustainable Nutrition

To answer the first research question, the results indicate that the in-service biology teachers who participated in interviews perceived a risk of indoctrination when teaching sustainable nutrition, primarily through their own actions in the classroom. Furthermore, vegan teachers were generally thought to be at risk of being more indoctrinating than omnivorous teachers. This finding is consistent with results from a pilot study with student biology teachers in Germany [38], as well as with studies showing that high school students attributed a higher indoctrination risk to vegan teachers, but at the same time perceived them as being more authentic [35,36].

Based on our findings, it can be derived that, in general, the perceived risk of indoctrination was pointed out as a “double-edged sword” (Mrs. Robinson, 45). The two edges of the sword illustrate two extremes that emerged from our results. One edge of the sword aligns with some participants, who were very open-minded and clearly stated that a certain degree of influencing students is a common part of teaching sustainable nutrition—however, this is done neither intentionally nor with any negative effects. A previous study even assumed that an important part of ESD is that teachers communicate sustainability-friendly values to their students [17]. Although people ultimately achieve sustainable development through concrete actions, ESD should not prescribe these actions—such as maintaining a vegan diet—to students, as this would be considered indoctrination [31]. However, teaching sustainable nutrition in the classroom should show the students that sustainable actions are necessary and possible [47].

The other edge of the sword represents the four teachers who seemed to be very cautious when teaching sustainable nutrition, in order to avoid indoctrinating their students—just as with sensitive topics such as sex or religious education. Moreover, some teachers were also cautious in their teaching because they felt they did not have sufficient pedagogical knowledge to teach ESD topics such as sustainable nutrition. In addition, three teachers seemed to feel very insecure about the methodological and didactical approaches to addressing this topic in the classroom. The fact that the participants were relatively cautious could also stem from the fact that they viewed themselves as role models, especially for young students. This is consistent with previous studies that have shown that teachers can be seen as role models by their students—especially regarding sustainable behavior [26]. In particular, the teachers in our study perceived an even higher risk of indoctrination when teaching younger students. The question, however, is whether it is solely positive if teachers are cautious when teaching sustainable nutrition. Being extremely cautious could result in insufficient or even no teaching of sustainable nutrition, as this topic is not part of the curricula. Thus, despite the potential of sustainable nutrition as an ESD topic, certain teachers may avoid teaching it simply to avoid worrying about indoctrination and about how to address their own beliefs in the classroom.

### 4.2. Research Question 2: Revealing One’s Own Dietary Style to Students

To answer the second research question, two participants related a higher risk of indoctrination to teachers with sustainable diets, such as vegetarian or vegan diets. Teachers can make students feel guilty about not behaving or eating sustainably to minimize their negative impact on their health and the environment. Mrs. Taylor, who was the only vegan teacher in our sample, perceived a risk of indoctrination due to her own dietary choices. According to her statements, she was very convinced about the health and environmental benefits of a vegan diet, and thus expressed strong beliefs about it—which she perceived as difficult to hide from students when teaching about sustainable nutrition in the classroom. According to Håkansson [27], teachers with especially strong beliefs about controversial topics—such as sustainable nutrition—should aim to remain almost neutral in their teaching of the topic.

Previous studies with high-school students have shown that they perceive vegan teachers as more authentic than omnivorous teachers when teaching about sustainable nutrition [35,36]. The students noted particular benefits from vegan teachers, such as teaching practical skills and relaying real-life experiences [35,36]. We conclude that in many cases, teachers seem to be more critical than students about the risk of indoctrination in the area of sustainable nutrition.

Two participants, Mr. Evans and Mr. Wood, were especially open-minded about bringing their personal dietary styles into the classroom. They mentioned the benefits of teaching sustainable nutrition to support students in investigating their own paths toward more sustainability. Additionally, they saw advantages in revealing their own dietary styles for building an appropriate student-teacher relationship, as well as for their own authenticity. Thus, these two teachers believed it is necessary to reveal their personal dietary styles to students. This perspective aligns with a prior study that showed home economics teachers often see the transfer of personal norms as an important part of their role as teachers [48].

Thus, two different perspectives have emerged to answer the second research question about revealing one’s own dietary style to students. One perspective includes the perceptions of teachers who argued for withholding their own dietary styles or only revealing them at students’ request, as they believed that teachers should generally hold back their own attitudes and beliefs. These participants and previous studies have both indicated that teachers, regarding their dietary styles, may be perceived as role models by their students [26].

The other perspective reflects those teachers who did not perceive a high risk of indoctrination when teaching sustainable nutrition. They were more open-minded toward reporting about their private-sphere lives and thus their personal dietary styles. For these participants, talking about their personal dietary styles was legitimate, authentic, and necessary in their function as role models. Being a role model in terms of sustainable eating behavior may entail some positive aspects, such as motivating and accompanying students who are interested in transforming their own dietary styles toward more sustainable nutrition.

Nevertheless, the teachers who expressed a more open-minded perspective would explain their students the reasons that lead them to behave in one way or another. In doing so, the participants want to ensure that they do not solely convey their own values to their students, which could then be regarded as indoctrination [30,31].

Teachers’ decisions to reveal their own dietary styles to their students—consequently being perceived as either authentic or indoctrinating—reveals a dilemma that biology teachers are likely to face when teaching sustainable nutrition. Whether in-service biology teachers who follow a sustainable dietary style—such as a vegan diet—are in fact more indoctrinating than teachers with an omnivorous lifestyle should be investigated in future studies.

### 4.3. Research Question 3: Approaches for Teaching Sustainable Nutrition and Avoiding Indoctrination

To answer the third research question, teachers described mostly student-centered methods and the need for including multiple perspectives in the classroom.

The participants advocated for a variety of student-centered methods, such as student-led discussions (e.g., “fishbowl discussion,” in which students in an inner circle discuss a topic from various viewpoints, with an outer circle of students who observe the discussion). In any case, the approaches should have a high level of student activity. Students should discover and evaluate the content of sustainable nutrition almost entirely on their own to minimize the teacher’s risk of indoctrinating their students. Methods such as the “fishbowl discussion” were considered by teachers to be suitable for teaching sustainable nutrition. These strategies aim to allow students to take their own position on a particular diet and compare it to other diets. In addition, participants expressed that the teacher should hold back here and allow students to lead the discussion, which may be beneficial for minimizing the risk of indoctrination [49], as many participants stated that teachers should be restrained in their own teaching actions when addressing sustainable nutrition, to avoid imposing their own beliefs on their students.

According to teachers in this study, biology teachers should support students to think critically and to make their own decisions in terms of sustainable nutrition. This is in line with the requirements of the educational mission of ESD in schools [19,50]. Multi-perspective biology teaching provides students with complementary perspectives on sustainable nutrition [51]. It broadens students’ perspectives, promotes a holistic grasp of sustainable nutrition, and is conducive for critical thinking skills [51]. Through multi-perspective approaches, the complexity and multi-faceted nature of sustainable nutrition—as well as the effects of one’s own dietary choices—become more transparent [51]. Thus, the complexity of sustainable nutrition cannot solely be addressed in lecture-based teaching or by conveying isolated content knowledge [52]. Moreover, multi-perspectivity is useful for younger students, as early as fifth grade [51], or for students for whom teachers saw a higher risk of indoctrination.

Furthermore, these findings indicate that biology teachers should provide multi-perspectivity when teaching sustainable nutrition to avoid indoctrinating. The results are consistent with the perceptions of high school students, who have been shown to desire an objective, multidimensional, and fact-based education for sustainable nutrition, in which they can form their beliefs independently of the teacher’s beliefs [35,36]. This corresponds to the controversy requirement of the Beutelsbach Consensus [32] and is an essential prerequisite for the development of evaluation competencies aligned with the educational mission of biology taught in schools [19,50].

### 4.4. Limitations of the Study

Due to the small sample size of the present study, it is not possible to make generalized statements about in-service biology teachers’ perceptions of a risk of indoctrination when teaching sustainable nutrition in classrooms [53]. In addition to the small sample size, a major limitation of the study is self-selection bias. As teachers were free to participate in the study, this may have biased the sample such that only teachers already interested in sustainable nutrition agreed to participate. Moreover, it is possible that the use of a digital interview influenced the results. Furthermore, Mrs. Williams initially had difficulties with the definition of the term “indoctrination”, which was relativized by providing the definition. This means, however, that her statements must be viewed critically. Despite the limitations, the study allows innovative insights and derives some educational implications for teaching sustainable nutrition.

## 5. Implications for Practice and Research

This study showed that our participants perceive a risk of indoctrination when teaching sustainable nutrition (research question 1) and that there are partly conflicting perceptions among our participants as to whether one should (or should not) be transparent about their own dietary style to students (research question 2). Additionally, the results show that teaching sustainable nutrition in biology lessons should primarily offer multi-perspective and student-centered approaches (research question 3).

As a main implication for practice, it can be derived that in-service biology teachers should learn how to deal with their own beliefs, attitudes, and behaviors when teaching topics such as sustainable nutrition against the backdrop of a potential risk of indoctrination. This can be addressed in in-service or already in university teacher training. Teacher training should provide in-service and student biology teachers with non-indoctrinating teaching approaches for implementing sustainable nutrition in biology classes. This may increase teachers’ self-efficacy toward teaching sustainable nutrition which can in turn promote their intention to teach sustainable nutrition [25].

Although the present study provided first insights into biology teachers’ ideas regarding an education for sustainable nutrition, it did not explore their actual classroom practices. Most of the participants stated during the interviews that—due to their educational mission—they should avoid indoctrination in the classroom. Future studies should examine more closely whether these statements correspond to the teachers’ actual classroom practices when teaching sustainable nutrition. Such a potential discrepancy between teachers’ beliefs and their actual classroom performance may be due to a lack of pedagogical knowledge about teaching sustainable nutrition [17]. Moreover, this discrepancy may be a result of the attitude-behavior gap, or the knowledge-behavior gap, or both [54,55]. In sum, practical experiences on how to avoid indoctrinating pedagogical practices should be part of seminars and lectures on teaching sustainable nutrition [17]. In these, challenges of teaching sustainable nutrition, such as being reflective about one’s own opinion and beliefs regarding sustainable nutrition, as well as explicit approaches for teaching sustainable nutrition, should be addressed to foster future and in-service biology teachers’ self-efficacy to teach sustainable nutrition without risking indoctrination.

## 6. Conclusions

To our knowledge, this is the first study to shed light on the teaching of sustainable nutrition in biology classes against the backdrop of a potential risk of indoctrination by focusing on personal beliefs, attitudes, and behaviors of biology teachers. This study is highly relevant, as choosing appropriate topics for addressing ESD in German biology lessons is mostly up to the teacher. Thus, if biology teachers feel competent and willing to teach sustainable nutrition, they may choose it as a topic for addressing ESD in biology classes. However, as their teaching actions may be influenced by their personal sustainability beliefs, attitudes, and behaviors, biology teachers need to be self-reflective enough to decide whether they are indoctrinating their students (or not) through their teaching actions and statements.

The findings of our study show that the participants perceived to be at risk of indoctrination when teaching sustainable nutrition, primarily due to teacher actions in the classroom. The results indicate that the participants held many different perceptions about the ways in which teaching sustainable nutrition is at risk of indoctrination. Participants’ ways of thinking were distributed between two extremes. One extreme indicates that teachers must be very restrained and careful when teaching sustainable nutrition, to avoid indoctrinating. The other extreme reflects teachers who are very open about revealing their own dietary style in the classroom for the benefit of students and see almost no risk of indoctrination.

Based on our findings, it can be summarized that the interviewed German biology teachers assigned high importance to multi-perspectivity when teaching sustainable nutrition: They mostly believed that it is not indoctrinating to reveal their own dietary style, as long as the teaching approaches involve multiple perspectives. This is consistent with the requirements of the competence-oriented educational mandate in terms of ESD [18,19] and with the “controversy requirement” of the Beutelsbach Consensus [31].

The findings of this study provide insights for researchers and teacher educators into biology teachers’ perceptions about teaching sustainable nutrition in classrooms and the potential risk of indoctrination. Thus, the results may provide valuable insights for further research as well as for designing seminars and lectures in biology teacher training, as further described in the Section 5.

## Figures and Tables

**Table 1:** Overview of the Biology Teacher Sample (*N* = 7).

Name	Mr. Smith	Mrs. Robinson	Mrs. Williams	Mrs. Wilson	Mr. Evans	Mrs. Taylor	Mr. Wood
Gender	male	female	female	female	male	female	male
Age	53 years	44 years	25 years	27 years	28 years	33 years	42 years
Teaching experience	21 years	20 years	2 years	1 year	2 years	7 years	14 years
Diet	omnivorous	pescatarian	vegetarian	omnivorous	vegetarian	vegan	flexitarian
ESD in school ^1^	+	+	++	+	+	++	++
SN in school ^1^	+	++	+	+	+	++	+
2nd/3rd subject	sports	German	sports	Latin	socialsciences	geography	geography/chemistry
School ^2^	1	2	3	4	5	6	6
Federal state ^3^	LS	LS	NRW	LS	NRW	NRW	NRW

Notes: ^1^ Participants were asked, “How important is it for you to address education for sustainable development (ESD) in everyday school life?” and “How important is the concrete topic of sustainable nutrition (SN)?” (++ = very important, + = important). ^2^ To maintain anonymity, the schools are indicated by numbers. Equal numbers mean that the teachers are employed at the same school. ^3^ LS = Lower Saxony, NRW = North Rhine-Westphalia. The names of the participants are replaced by pseudonyms.

**Table 2:** Summary of the Main Findings Regarding the Three Research Questions.

Teacher	Research Question 1	Research Question 2	Research Question 3
	Risk of Indoctrination	Sharing Own Dietary Style with Students	Approaches to Teaching Sustainable Nutrition
Mr. Smith	Yes; primarily due to learning materials; teachers are responsible for avoiding indoctrination	No, only fact-based and neutral, thus no risk of indoctrination	Project-based, primarily knowledge transfer
Mrs. Robinson	Yes; depending on teachers’ actions and beliefs; primarily due to teachers’ role model function—especially for younger students	Yes, as students are interested	Project-based, basic terms and concepts, field trips, multi-perspectivity
Mrs. Williams	Partly; depending on teachers’ actions and beliefs; withholding own opinions may be difficult for teachers—especially for environmentally conscious teachers	Only on students’ demand, no risk of indoctrination	Project-based, multi-perspectivity
Mrs. Wilson	Yes; depending on teachers’ actions and beliefs	Generally yes, but depending on the group of students; potential conflict between role model function and indoctrination	Evaluation competencies, project-based, discussions
Mr. Evans	Yes; depending on teachers’ actions; especially when teaching younger students	Only on students’ demand, but if students ask then of course, as it is part of an open and personal relationship with the students	Group work, multi-perspectivity
Mrs. Taylor	Yes; depending on teachers’ actions and beliefs; withholding own opinion may be difficult for teachers—especially when being vegan; experienced risk of indoctrination herself	Only on students’ demand; students are interested, potential risk of indoctrination, especially due to her own vegan dietary style	Multi-perspectivity
Mr. Wood	Generally yes; depending on teachers’ actions	Yes, as it is part of teachers’ authenticity; at risk of indoctrination	Multi-perspectivity, field trips, evaluation competencies, student-centered

## Data Availability

The data can be obtained from the corresponding author upon request.

## Supplementary Materials

The following supporting information can be downloaded at: https://www.mdpi.com/article/10.3390/foods11060887/s1, File S1: Semi-Structured Interview Guide.
