# Supplementary material for: “Vegan Teachers Make Students Feel Really Bad”: Is Teaching Sustainable Nutrition Indoctrinating?
Source: Foods. 2022 Mar 21;11(6):887. doi: 10.3390/foods11060887 (PMC8954596; doi:10.3390/foods11060887)
Supplement: Supplementary file 1 [file foods-11-00887-s001.zip › foods-1623612-supplementary.pdf]

## Supplementary Material File S1: Semi-Structured Interview Guide

| **Card:** 1 | **Section:** 1 - Small Talk | **Intention/ research interest:** Relax the situation, start the interview |
|---|---|---|

**Preparations:**

- Turn on recording device (prepare MacBook as backup for voice recording).
- Establish video connection
- Have interview guide, a piece of paper, and a pencil ready

**Procedure:**

- Welcome
- Fix technical problems if necessary
- Short small talk
    - Can you understand and see me? […]
- Clarify schedule: Is there a time frame to meet due to subsequent deadlines? The interview takes approx. 30 minutes

| **Card:** 2 | **Section:** 2 – Information on the interview | **Intention/ research interest:** Clarification about interview procedure |
|---|---|---|

**Procedure:**

- I'd like to give you some information about the interview procedure and how I'm planning to use the information.
- I'll just start with me: I am (name) and I am a researcher in biology didactics.
- Today I would like to talk with you about sustainable nutrition as a teaching topic and its potential risk of indoctrination.
- I am not interested in your knowledge about sustainable nutrition, but in your experiences as a biology teacher.
- I will record this interview with a recording device (see also privacy policy). The recording will be typed up afterwards and then deleted, as we will continue working with the written document. I will anonymize everything related to this interview. Neither your name nor the school you teach at will be mentioned.
- Your participation in the interview is voluntary. You can stop or leave a question unanswered at any time.
- After the interview, I would like to fill out a short questionnaire together with you on general information about your person.
- Do you have any questions?
- If not, I would now start recording. Is that all right? (Start screen recording and audio device)

<table>
<tr><td>Card: 3</td><td colspan="2">Section: 3 – Introduction, Part 1<br>Topic: Associations and conceptions of sustainable nutrition</td><td>Intention/ research interest:<br>Thematic introduction<br>Associations with sustainable nutrition</td></tr>
<tr><td colspan="4">Guiding question/ narrative prompt:<br>My first question is more general in nature: What associations do you have with sustainable nutrition? Please write down at least five terms that come to your mind first.</td></tr>
<tr><td colspan="4">Alternative phrase(s):<br>- When you hear the term “sustainable nutrition”, what (at least) five terms come to your mind spontaneously</td></tr>
<tr><td>Content aspects<br>• Respondents writes down at least 5 terms<br>• Explanation of the terms and other aspects (meaning, practical implementation, etc.)<br>• Personal conceptions of sustainable nutrition</td><td>Further questions/ remarks:<br>• Could you give me your personal definition of sustainable nutrition?<br>• Go through the terms one by one and have the respondents explain each in their own words.<br>Possible follow-up questions about the terms:<br>• How did you come up with this?</td><td>Intervention/ other:<br>If respondents ask for solutions, "I'll be happy to make a note and then we'll talk about it at the end."<br>Possibility: Giving definition of sustainable nutrition</td><td>Expectations horizon:<br>Proportions of the 5 dimensions: Culture, Economy, Environment, Health, Society<br>fruit/ vegetables; $CO_2$ emissions; organically grown food; diets (vegan/vegetarian); regional/ seasonal food</td></tr>
</table>

<table>
<tr><td>Card: 4</td><td colspan="2">Section: 4 – Main part, Part 2<br>Topic: Classroom implementation of sustainable nutrition in biology lessons</td><td>Intention/ research interest:<br>How is sustainable nutrition taught?</td></tr>
<tr><td colspan="4">Guiding question/ narrative prompt:<br>• Have you already addressed the topic of sustainable nutrition in biology lessons?<br>• If yes, how did you approach the topic? What did you pay attention to?<br>• If no, how would you implement the topic in biology lessons?</td></tr>
<tr><td colspan="4">Alternative phrase(s):</td></tr>
<tr><td>Content aspects:<br>• 5 dimensions of sustainable nutrition<br>• Definition sustainable nutrition<br>• Influencing students Disregard own opinion</td><td>Further questions/ remarks:<br>• What methods would you use to teach sustainable nutrition?<br>• What aspects are/were important for you when teaching sustainable nutrition?<br>• What content was included/should be included?<br>• How would you embed the topic in biology lessons (Keyword: ESD or nutrition)?<br><u>If already taught:</u><br>Have you taught sustainable nutrition due to personal interest?</td><td>Intervention/ other:</td><td>Expectations horizon:<br>Aspects of the dimensions and principles according to von Koerber (2014); embedding in contexts of SDGs/ environmental protection/biodiversity/ESD; data-based decision making; group discussions; methods that enable multi-perspectivity; evaluation competencies</td></tr>
<tr><td colspan="4">Additional information: Definition of sustainable diets<br>Sustainable diets have a low impact on the environment, contribute to food and nutrition security, and enable current and future generations to live healthy lives. They protect and respect biodiversity and ecosystems, are culturally appropriate, available, economically equitable and affordable, safe and healthy, while enhancing natural and human livelihoods.<br><br>Source: FAO (Food and Agriculture Organization of the United Nations) (2012). In Burlingame B. & Dernini S. (eds). Sustainable diets and biodiversity - Directions and solutions for policy research and action. Proceedings of the International Scientific Symposium Biodiversity and Sustainable Diets United Against Hunger. FAO, Rome.<br>Sustainable diets therefore mean eating in a way that maximizes the overall health, environmental, economic, cultural and social impacts of our diet. For example, by consuming fair trade, regional and/or organic foods.</td></tr>
</table>

<table>
<tr><td>Card: 5</td><td colspan="2">Section: 5 – Main part, part 3<br>Topic: Indoctrination as problem when teaching sustainable nutrition?</td><td>Intention/ research interest:<br>Field of tension: Teaching sustainable nutrition at risk of indoctrination</td></tr>
<tr><td colspan="4">Guiding question/ narrative prompt:<br>• Do you perceive a risk of indoctrination when teaching sustainable nutrition?<br>• If yes, why? If no, why not?<br>• What explicit methods and approaches would you use to avoid indoctrination?</td></tr>
<tr><td colspan="4">Alternative phrase(s):<br>• What does teachers need to do to avoid indoctrinating their students?<br>• In what aspects must a teacher be especially careful not to indoctrinate?</td></tr>
<tr><td>Content aspects<br>• Own experiences<br>• indoctrination</td><td>Further questions/ remarks:<br>Are the methods similar to those mentioned above?<br>Would you reveal you own dietary styles to your students? Why? Why not? To what extent?</td><td>Intervention/ other:<br>• ask for possible teaching approaches</td><td>Expectations horizon:<br>Integrating the own opinion too strong; Risk of indoctrination due to lack of content knowledge</td></tr>
</table>

<table>
<tr><td>Card: 6</td><td>Section: Closing</td><td>Intention:<br>Conclusion of the interview, clarify questions, additions</td></tr>
<tr><td colspan="3">Procedure:<br>• I am done with my questions so far. Do you have anything else you would like to add? Do you have any questions?<br>• Were there any difficulties in understanding or other problems during the interview? Did you find things particularly difficult or did things bother you? Through your feedback I could possibly optimize something for the following interviews<br>• Filling out the questionnaire together<br>• End of recording - MacBook and recording device<br>• Great, thank you. Then I now have everything I need.<br>• Thank you very much for taking part. I hope it was a little fun for you.<br>• Stay healthy! (Interviewer finishes filling out the questionnaire)</td></tr>
</table>
